# Risk factors associated with functional dyspepsia in Chinese children: a cross-sectional study

**DOI:** 10.1186/s12876-021-01800-x

**Published:** 2021-05-12

**Authors:** Zhongcao Wei, Xing Yang, Xin Xing, Lei Dong, Jinhai Wang, Bin Qin

**Affiliations:** 1 Department of Gastroenterology, The Second Affiliated Hospital, Xi’an Jiaotong University, Xi’an, Shaanxi China; 2 Department of General Medicine, The Second Affiliated Hospital, Xi’an Jiaotong University, Xi’an, Shaanxi China; 3 Department of Cardiology, The Second Affiliated Hospital, Xi’an Jiaotong University, Xi’an, Shaanxi China

**Keywords:** Functional dyspepsia, Risk factors, Chinese children, Rome IV

## Abstract

**Background:**

There is no study assessing the risk factors associated with functional dyspepsia (FD) in Chinese children based on the Rome IV criteria.

**Methods:**

In this cross-sectional study, we analyzed data from eight representative primary and secondary schools to assess the risk factors associated with FD in Chinese children based on the Rome IV criteria.

**Results:**

A total of 6976 Chinese children were enrolled. The mean age was 14.3 ± 2.5 years, with a range from 7 to 17 years, and 3497 (50.1%) participants were female. FD was prevalent in 209 (3.0%) of the Chinese child population studied. Age (OR = 1.112, *P* = 0.006), living independent of parents (OR = 1.677, *P* < 0.001), prolonged school meals (OR = 2.107, *P* < 0.001), never eat breakfast (OR = 2.192, *P* = 0.003), often/daily eat cold foods (OR = 2.296, *P* = 0.002; OR = 2.736, *P* = 0.011), and often eat pickled foods (OR = 2.390, *P* = 0.001) were found to be independent risk factors for FD. A nomogram with these risk factors had good discrimination (AUC = 0.727) and calibration (Hosmer–Lemeshow test was 0.851).

**Conclusions:**

Age, living independent of parents, prolonged school meals, never eat breakfast, often/daily eat cold foods and often eat pickled foods were independent risk factors for FD. The nomogram could be used as a quick screening tool to assess FD in Chinese children.

## Background

Functional dyspepsia (FD) is a gastrointestinal (GI) disorder syndrome characterized by symptoms such as abdominal pain, epigastric burning, postprandial fullness or early satiation [1–3]. The prevalence of FD has been estimated to range from < 2% to 57% in the general worldwide population [4–7]. Although FD is rarely fatal, it is associated with a significant reduction in the quality of life, has a significant, negative impact on work efficiency and increases medical costs for the patient and society [8–10].

Current treatments for FD are unsatisfactory for both physicians and patients, and few treatment options have been shown to be effective against FD thus far [11, 12]. Many FD patients report that dyspeptic symptoms are associated with dietary habits. Diet-related risk factors in populations with FD have been largely unexplored [13, 14]. There is a lack of relevant research data in the Chinese population, and risk factors in the Chinese population need further study.

The diagnosis of FD is clinical and based on the Rome criteria. The Rome IV criteria were introduced in 2016 as an update of the Rome III criteria [15, 16]. For children, the Rome IV criteria have been modified in terms of the time required to diagnose FD (at least four days a month) and in the established subcategories (dividing FD into postprandial distress syndrome and epigastric pain syndrome) [17, 18]. There have been few studies assessing FD based on the Rome IV criteria, especially among Chinese children; hence, further studies assessing FD based on the Rome IV criteria are urgently needed.

Understanding the risk factors of FD on the basis of Rome IV criteria will contribute to the diagnosis and prevention of FD. The aim of this study was to assess the diet-related risk factors associated with FD based on the Rome IV criteria and to identify other risk factors associated with FD in Chinese children.

## Methods

### Study design

This cross-sectional study was conducted at eight representative primary and secondary schools to assess the risk factors associated with FD in Chinese children based on the Rome IV criteria in Shaanxi province, China, from March 2017 to September 2017. All procedures were conducted in accordance with the relevant guidelines and regulations. Patients who met the inclusion and exclusion criteria were included in the analysis. The inclusion criteria included all children (ages 7 to 17 years) in each school. And the exclusion criteria were not willing to participate in the study, age ≥ 18 years or incomplete data. Written informed consent was obtained from school administrators and the participating children prior to the survey. This study was approved by the Ethics Committee of the Second Affiliated Hospital of Xi’an Jiaotong University.

### Sample size calculation

Sample size calculation was based on point that the prevalence of FD has been estimated to range from < 2 to 57% in the general worldwide population [4–7].

We selected the lower incidence rate of 3% as the reference standard, with a 0.6% margin of error and 95% confidence level. The sample size calculated by the PASS 15.0 software was 3277. Assuming that the pass rate of the questionnaire is 80%, at least 3933 children needed to be screened for this study.

### Data collection

All related data were collected through questionnaires according to the Rome IV criteria. Face-to-face interviews were conducted with all participating children, and all the interviews were carried out by trained field investigators. Basic demographic data included name, gender, age, height, and weight, height and weight were used to calculate the body mass index (BMI). We also collected growth history (history of breastfeeding, living independent of parents, long school accommodations (school accommodations ≥ five days per week) and prolonged school meals (school meals ≥ five days per week)), dyspeptic information (dyspeptic symptoms, symptom durations, symptom frequencies and the relationship with defecation) and dietary habits (breakfast, staple foods, cold foods, spicy foods, milk products, carbonated beverages, high-fiber foods, fried foods, salty foods, desserts, highly starchy foods, fruits, vegetables). The dietary frequency was divided into never, occasionally (1–2 times per week), sometimes (3–4 times per week), often (more than 5 times a week, but not every day), and daily [19]. And each question was explained to the participating children in detail to help them understand by the investigators.

### Definition of FD

The presence or absence of FD was determined by the questionnaire according to the Rome IV criteria. FD was defined as at least one of postprandial fullness, early satiety, epigastric pain or epigastric burning that occurred for at least 2 months and at least 4 days in each month. Epigastric pain syndrome (EPS) was defined as at least one of epigastric pain or epigastric burning that occurred for at least 2 months and at least 4 days in each month. Postprandial distress syndrome (PDS) was defined as at least one of postprandial fullness or early satiety that occurred for at least 2 months and at least 4 days in each month.

### Statistical analysis

Statistical analyses were conducted using SPSS 20.0 software (IBM Corp., Armonk, New York, USA) and R software (version 4.0.1). Categorical variables are reported as counts and percentages and were evaluated using chi-square tests or Fisher’s exact test when appropriate. Continuous variables are reported as the means ± SDs and were evaluated using a *t* test or the Kruskal–Wallis test when appropriate. All variables were explored by univariate analysis, and variables with *P* values < 0.10 in univariate analysis were entered into the multivariate logistic regression analysis. Data were expressed as ORs and 95% confidence intervals (CIs). Statistical significance was defined as *P* < 0.05. A nomogram was constructed based on the logistic regression results. The discrimination was evaluated using the area under receiver operating characteristic (ROC) curve, and calibration was evaluated using the calibration curve and the Hosmer–Lemeshow test.

## Results

### Baseline of patient characteristics

**Fig. 1:**
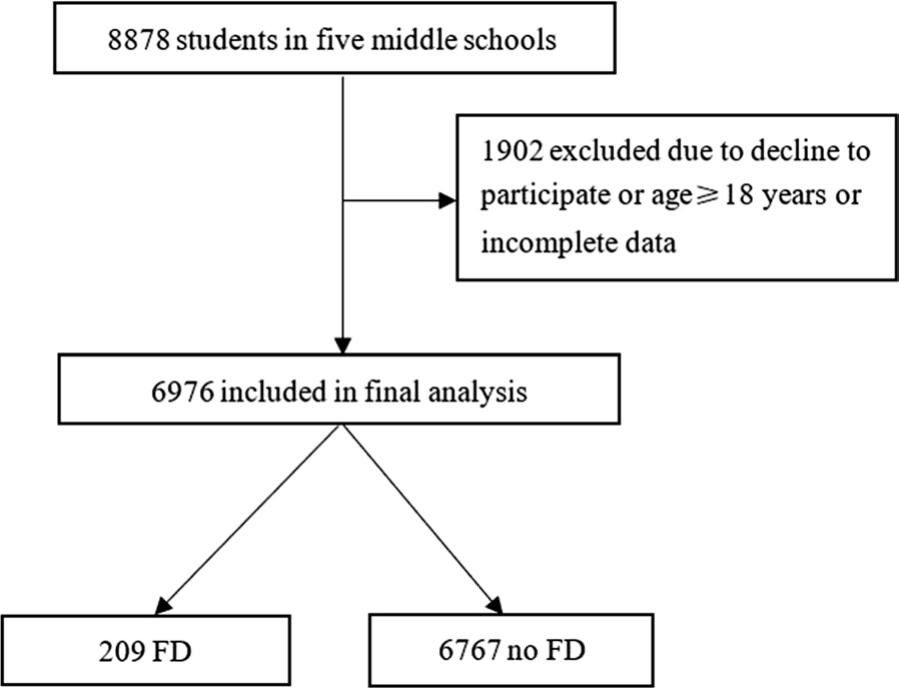
Flowchart of the study. *FD* functional dyspepsia

We screened 8878 students from eight primary and secondary schools; after excluding 1902 students due to decline to participate or age ≥ 18 years or incomplete data, a total of 6976 school students were included in the final analysis, including 209 with FD and 6767 without. The flowchart of the study is shown in Fig. 1. The mean age was 14.3 ± 2.5 years, with a range from 7 to 17 years, and 3497 (50.1%) participants were female. The majority of the population 5978 (85.7%) had a history of breastfeeding, and a total of 2773 (39.8%) lived independent of parents. In all, 3315 (47.5%) children had long school accommodations, and 3881 (55.6%) had prolonged school meals (Table 1).

**Table 1:** Univariate and multivariate analyses of functional dyspepsia (FD) and no FD

Characteristics	No FD (n = 6767)	FD (n = 209)	*P* value^a^	Multivariate analyses		
				OR	95%CI	*P* value^b^
Age	14.3 ± 2.5	15.3 ± 2.1	< 0.001	1.112	1.031–1.201	0.006
BMI	19.9 ± 3.7	20.1 ± 3.4	0.054			
Sex			0.086			
Male	3387 (50.1)	92 (44.0)				
Female	3380 (49.9)	117 (56.0)				
History of breastfeeding			0.825			
Yes	5800 (85.7)	178 (85.2)				
No	967 (14.3)	31 (14.8)				
Living independent of parents			< 0.001			
Yes	2650 (39.2)	123 (58.9)		1.677	1.255–2.242	< 0.001
No	4117 (60.8)	86 (41.1)		1.00		
Long school accommodation			< 0.001			
Yes	3166 (46.8)	149 (71.3)				
No	3601 (53.2)	60 (28.7)				
Prolonged school meals			< 0.001			
Yes	3715 (54.9)	166 (79.4)		2.107	1.447–3.068	< 0.001
No	3052 (45.1)	43 (20.6)		1.00		
Staple foods			< 0.001			
Mainly noodles	679 (10.0)	19 (9.1)				
Mainly rice	1743 (25.8)	78 (37.3)				
Rarely eat	299 (4.4)	15 (7.2)				
Both	4046 (59.8)	97 (46.4)				
Breakfast			< 0.001			0.005
Never	262 (3.9)	23 (11.0)		2.192	1.303–3.688	0.003
Occasionally	1139 (16.8)	43 (20.6)		1.014	0.671–1.530	0.948
Sometimes	864 (12.8)	31 (14.8)		1.065	0.679–1.673	0.783
Often	2190 (32.4)	53 (25.4)		0.783	0.535–1.148	0.211
Daily	2312 (34.2)	59 (28.2)		1.00		
Cold foods			< 0.001			< 0.001
Never	1038 (15.3)	26 (12.4)		1.00		
Occasionally	3606 (53.3)	84 (40.2)		0.866	0.549–1.367	0.538
Sometimes	1543 (22.8)	47 (22.5)		1.019	0.615–1.688	0.942
Often	467 (6.9)	40 (19.1)		2.296	1.347–3.912	0.002
Daily	113 (1.7)	12 (5.7)		2.736	1.263–5.927	0.011
Spicy foods			< 0.001			
Never	1187 (17.5)	34 (16.3)				
Occasionally	3244 (47.9)	86 (41.1)				
Sometimes	1731 (25.6)	50 (23.9)				
Often	502 (7.4)	32 (15.3)				
Daily	103 (1.5)	7 (3.3)				
Milk products			0.250			
Never	390 (5.8)	17 (8.1)				
Occasionally	1243 (18.4)	30 (14.4)				
Sometimes	1733 (25.6)	53 (25.4)				
Often	1913 (28.3)	55 (26.3)				
Daily	1488 (22.0)	54 (25.8)				
Carbonate beverages			0.753			
Never	1761 (26.0)	53 (25.4)				
Occasionally	2815 (41.6)	83 (39.7)				
Sometimes	1339 (19.8)	40 (19.1)				
Often	653 (9.6)	25 (12.0)				
Daily	199 (2.9)	8 (3.8)				
High–fiber foods			0.019			
Never	1196 (17.7)	46 (22.0)				
Occasionally	2360 (34.9)	88 (42.1)				
Sometimes	1835 (27.1)	40 (19.1)				
Often	981 (14.5)	27 (12.9)				
Daily	395 (5.8)	8 (3.8)				
Fried foods			< 0.001			
Never	1020 (15.1)	30 (14.4)				
Occasionally	3120 (46.1)	65 (31.1)				
Sometimes	1765 (26.1)	63 (30.1)				
Often	688 (10.2)	38 (18.2)				
Daily	174 (2.6)	13 (6.2)				
Pickled foods (n, %)			< 0.001			0.010
Never	2382 (35.2)	63 (30.1)		1.00		
Occasionally	2864 (42.3)	80 (38.3)		1.032	0.731–1.456	0.860
Sometimes	1134 (16.8)	36 (17.2)		1.018	0.660–1.571	0.935
Often	312 (4.6)	25 (12.0)		2.390	1.435–3.981	0.001
Daily	75 (1.1)	5 (2.4)		1.368	0.485–3.859	0.553
Dessert (n, %)			0.013			
Never	788 (11.6)	17 (8.1)				
Occasionally	2542 (37.6)	68 (32.5)				
Sometimes	1918 (28.3)	57 (27.3)				
Often	1150 (17.0)	48 (23.0)				
Daily	369 (5.5)	19 (9.1)				
High starchy foods (n, %)			0.215			
Never	432 (6.4)	20 (9.6)				
Occasionally	1503 (22.2)	48 (23.0)				
Sometimes	2284 (33.8)	58 (27.8)				
Often	1791 (26.5)	60 (28.7)				
Daily	757 (11.2)	23 (11.0)				
Fruits (n, %)			0.068			
Never	199 (2.9)	11 (5.3)				
Occasionally	1135 (16.8)	46 (22.0)				
Sometimes	1726 (25.5)	52 (24.9)				
Often	2011 (29.7)	55 (26.3)				
Daily	1696 (25.1)	45 (21.5)				
Vegetables (n, %)			0.090			
Never	230 (3.4%)	11 (5.3)				
Occasionally	931 (13.8%)	38 (18.2)				
Sometimes	1519 (22.4)	50 (23.9)				
Often	1952 (28.8)	58 (27.8)				
Daily	2135 (31.6)	52 (24.9)				

Values are expressed as the means ± SDs or n (%)

*BMI* body mass index, *M* male, *F* female

^a^*P* value of univariate

^b^*P* value of multivariate analyses

### Prevalence of FD in children

**Fig. 2:**
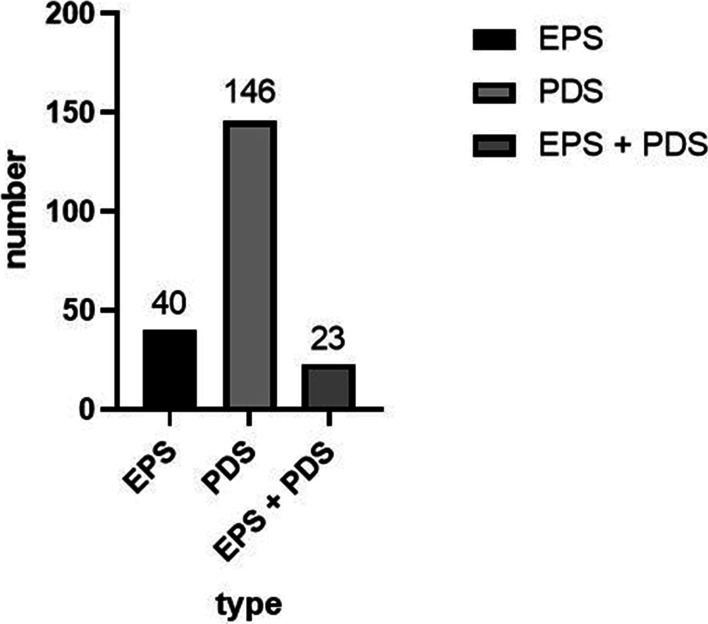
Symptom subtypes of functional dyspepsia (FD) according to the Rome IV criteria. *EPS* epigastric pain syndrome; *PDS* postprandial discomfort syndrome

FD, according to the Rome IV criteria, was prevalent in 209 (3.0%) of the Chinese child population studied. Among the 209 children with FD, the mean age was 15.3 ± 2.1 years, and 117 (56.0%) of the sufferers were female. Among the children with FD according to the Rome IV criteria, 40 children only presented with EPS, 146 children only met the criteria for PDS, and 23 children presented with both EPS and PDS. The symptom subtypes of FD in this population are shown in Fig. 2.

### Risk factors for FD in children

Comparisons of various factors between the children with FD and those without (Table 1). In univariate analysis, there were statistically significant differences between FD and no FD in age, living independent of parents, long school accommodation, prolonged school meals, staple foods, breakfast, cold foods, spicy foods, high-fiber foods, fried foods, pickled foods (*P* < 0.001), and desserts (*P* = 0.013).

The independent risk factors for FD in Chinese children were explored by a multivariate logistic regression analysis. Any variables with *P* values < 0.10 in the univariate analysis were incorporated into multivariate analysis. In the multivariate logistic regression analysis, BMI, gender, fruits, vegetables were analyzed together with age, living independent of parents, long school accommodations, prolonged school meals, staple foods, breakfast, cold foods, spicy foods, high-fiber foods, fried foods, pickled foods, desserts. Age (OR = 1.112, 95% CI = 1.031–1.201, *P* = 0.006), living independent of parents (OR = 1.677, 95% CI = 1.255–2.242, *P* < 0.001), prolonged school meals (OR = 2.107, 95% CI = 1.447–3.068, *P* < 0.001), never eat breakfast (OR = 2.192, 95% CI = 1.103–3.688, *P* = 0.003), often/daily eat cold foods (OR = 2.296, 95% CI = 1.347–3.912, *P* = 0.002; OR = 2.736, 95% CI = 1.263–5.927, *P* = 0.011), and often eat pickled foods (OR = 2.390, 95% CI = 1.435–3.981, *P* = 0.001) were discovered to be the independent risk factors for FD (Table 1).

### Creation of the nomogram

**Fig. 3:**
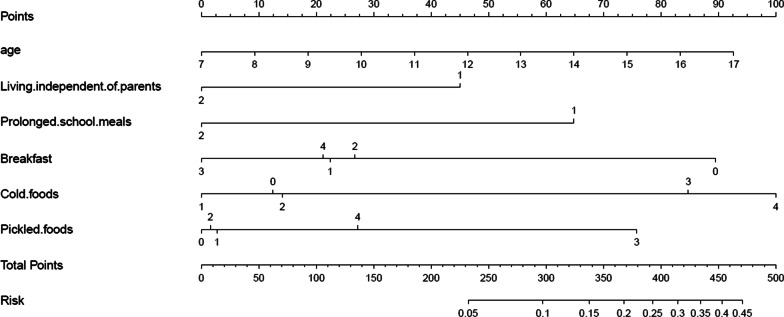
A nomogram for predicting risk of functional dyspepsia (FD) from no FD. For binary variables (living independent of parents, prolonged school meals), 2 = no and 1 = yes. For multiple categorical categories (breakfast, cold foods, pickled foods), 0 = never, 1 = occasionally, 2 = sometimes, 3 = often, and 4 = daily

**Fig. 4:**
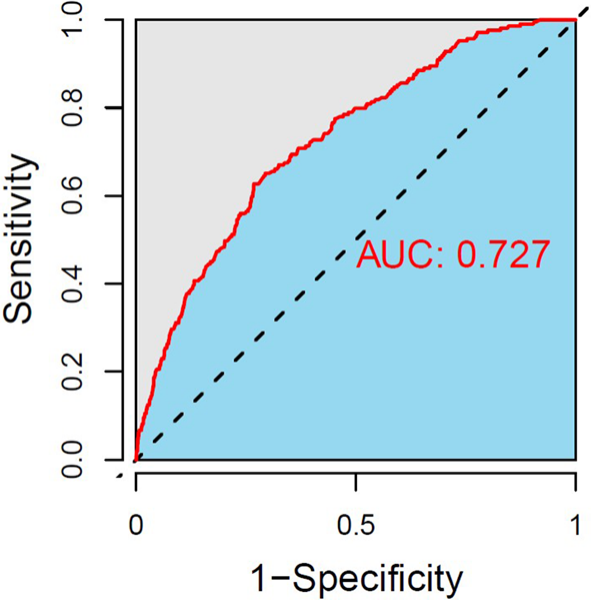
ROC curves of the nomogram

**Fig. 5:**
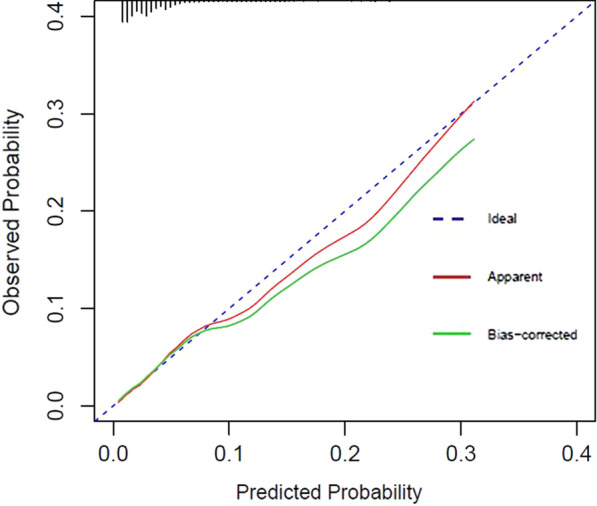
Calibration curves of the nomogram

Based on the independent risk factors of FD, a nomogram was established (Fig. 3). The variables independently associated with FD and included in the final nomogram were age, living independent of parents, prolonged school meals, breakfast, cold foods, pickled foods. At the top of the nomogram was a reference line that represented the score from 0 to 100 for each variable. By summing the total score of each predictor, the probability of FD could be estimated effectively. The predictive value of the nomogram was assessed by the ROC curve, calibration curve and Hosmer–Lemeshow test. The area under the curve (AUC) was 0.727, showed good discrimination (Fig. 4). The calibration curve was close to the diagonal line (Fig. 5) and the Hosmer–Lemeshow test was 0.851 > 0.05, showing good calibration.

## Discussion

As far as we know, this study was the first study to assess the risk factors associated with FD in Chinese children based on the Rome IV criteria. As FD is considered a chronic recurrent disease and has seriously reduced the quality of life and significantly increased healthcare costs, studies have focused on searching for the causes of and treatment methods for FD [20–22]. In this cross-sectional study, we assessed the diet-related risk factors and other risk factors associated with FD based on the Rome IV criteria, and found that age, living independent of parents, prolonged school meals, never eat breakfast, often/daily eat cold foods, and often eat pickled foods were independent risk factors for FD. Moreover, we developed a diagnostic nomogram to assess FD based on the Rome IV criteria. The total score can be calculated by collecting the information of the variables on the nomogram of every Chinese children.

In our study, among the 6976 study participants, the proportion with FD was 3.0% (209/6976) among the Chinese children. A study of FD in Japan demonstrated that after screening 2060 children using the Rome III criteria, the proportion of children who had FD was 2.8% [23]. A previous cross-sectional study involving 2136 children with functional gastrointestinal diseases showed that 2.5% (54/2136) of the children met the Rome III criteria for FD [24]. Among other studies of FD in children, the proportion who met the Rome III criteria for FD was between 0.9% and 16% [25–27]. Overall, the proportion of FD in our study was remarkably comparable to those of other studies.

Although a majority of patients with FD associate dyspeptic symptoms with dietary habits, studies on diet-related factors in FD have been limited, and the results associating diet-related factors and FD are conflicting [28–31]. Wang et al. showed that cold stimulation could increase intragastric pressure in patients, elevating visceral sensitivity and reducing the gastric volume of patients. These observations were consistent with our study results that cold foods were an independent risk factors for FD [32]. In our study, children with FD were slightly older than children without, which is in line with the results of previous studies [28]. We identified that children in this population living independent of parents, who took prolonged school meals, never eat breakfast, and often eat pickled foods were more likely to have FD. However, scientific evidence for studying the efficacy of dietary and other related factors in FD is still lacking. Therefore, further well-designed studies are still needed to establish dietary risk factors related to FD.

In our study, although living independent of parents, prolonged school meals, never eat breakfast, often/daily eat cold foods, often eat pickled foods were the independent risk factors for FD, these factors had close relationship with each other. Living independent of parents was associated with bad eating habits. Children living independent of parents will largely face risk factors such as prolonged school meals, often/daily eat cold foods, so living independent of parents was a basic and very important factor among these risk factors. In the future, more attention should be paid to children living independent of parents.

The main strength of our study is that it was a relatively large cross-sectional study designed to examine diet-related and other risk factors in Chinese children with FD based on the current Rome IV criteria. In contrast, this study also had several limitations. First, the samples in our study are all from schools in Shaanxi province and are therefore a small representation of Chinese children. There may be regional differences between our sample and other children throughout China; thus, the results of our study may not reflect the reality of the general Chinese child population, which may influence the external validity of the research results. In addition, the study population we included did not undergo gastroscopy, so we could only analyze the risk factors related to FD and could not further analyze those related to functional and organic FD.

## Conclusions

Age, living independent of parents, prolonged school meals, never eat breakfast, often/daily eat cold foods, and often eat pickled foods were independent risk factors for FD in Chinese children based on the Rome IV criteria. The nomogram could be used as a quick screening tool to assess FD in Chinese children. This may contribute to the prevention and treatment options of FD.

## Data Availability

The datasets used and/or analyzed during the current study are available from the corresponding author on reasonable request.

## Abbreviations

AUC: Area under the curve
BMI: Body mass index
CI: Confidence interval
EPS: Epigastric pain syndrome
F: Female
FD: Functional dyspepsia
GI: Gastrointestinal
M: Male
PDS: Postprandial discomfort syndrome
ROC: Receiver operating characteristic
